# Examining geographic and socio-economic differences in outpatient and inpatient consumer expenditures for treating HIV/AIDS in Nigeria

**DOI:** 10.7448/IAS.19.1.20588

**Published:** 2016-02-01

**Authors:** Obinna E Onwujekwe, Ogochukwu Ibe, Kwasi Torpey, Stephanie Dada, Benjamin Uzochukwu, Olusola Sanwo

**Affiliations:** 1 Department of Pharmacology and Therapeutics, College of Medicine, University of Nigeria Enugu Campus, Enugu, Nigeria; 2 Department of Health Administration and Management, College of Medicine, University of Nigeria Enugu Campus, Enugu, Nigeria; 3 Health Policy Research Group, College of Medicine, University of Nigeria Enugu Campus, Enugu, Nigeria; 4 Department of Health Systems, FHI 360, Abuja, Nigeria; 5 Department of Community Medicine, College of Medicine, University of Nigeria Enugu Campus, Enugu, Nigeria

**Keywords:** HIV/AIDS, ART services, inequity, economic burden, catastrophic health expenditure

## Abstract

**Introduction:**

The expenditures on treatment of HIV/AIDS to households were examined to quantify the magnitude of the economic burden of HIV/AIDS to different population groups in Nigeria. The information will also provide a basis for increased action towards a reduction of the economic burden on many households when accessing antiretroviral therapy (ART).

**Methods:**

A household survey was administered in three states, Adamawa, Akwa Ibom and Anambra, from the South-East, North-East and South-South zones of Nigeria, respectively. A pretested interviewer-administered questionnaire was used to collect data from a minimum sample of 1200 people living with HIV/AIDS (PLHIV). Data were collected on the medical and non-medical expenditures that patients incurred to treat HIV/AIDS for their last treatment episode within three months of the interview date. The expenditures were for outpatient visits (OPV) and inpatient stays (IPS). The incidence of catastrophic health expenditure (CHE) on ART treatment services was computed for OPV and IPS. Data were disaggregated by socio-economic status (SES) and geographic location of the households.

**Results:**

The average OPV expenditures incurred by patients per OPV for HIV/AIDS treatment was US$6.1 with variations across SES and urban-rural residence. More than 95% of the surveyed households spent money on transportation to a treatment facility and over 70% spent money on food for OPV. For medical expenditures, the urbanites paid more than rural dwellers. Many patients incurred CHE during outpatient and inpatient visits. Compared to urban dwellers, rural dwellers incurred more CHE for outpatient (*p*=0.02) and inpatient visits (*p*=0.002).

**Conclusions:**

Treatment expenditures were quite high, inequitable and catastrophic in some instances, hence further jeopardizing the welfare of the households and the PLHIV. Strategically locating fully functional treatment centres to make them more accessible to PLHIV will largely reduce expenditures for travel and the need for food during visits. Additionally, financial risk-protection mechanisms such as treatment vouchers, reimbursement and health insurance that will significantly reduce the expenditures borne by PLHIV and their households in seeking ART should be implemented.

## Introduction

An estimated 3.1 million people are living with HIV/AIDS in Nigeria, and the epidemiological distribution indicates significant diversity across the country's geographic settings [1]. In 2008, the national sero-prevalence rate of HIV/AIDS was 4.6% with a corresponding decline to 4.1 and 3.4% in 2010 and 2013, respectively [1,2]. As with preceding years, the 2013 report shows that the prevalence was higher among wealthier Nigerians (3.7%) than among poorer Nigerians (2.9%). There were similar findings when the rural dwellers (3.6%) were compared with the urban dwellers (3.2%) [2]. Based on projected HIV estimates, 220,394 new HIV infections occurred in 2013, a total of 210,031 died from AIDS-related causes and an estimated 1,476,741 required antiretroviral (ARV) drugs in 2013 [3].

In Nigeria, ARV drugs are free of charge to patients at designated health facilities, and the provision of free drugs has apparently improved access to ARVs across the country [4]. However, because HIV/AIDS increases vulnerability to other illnesses, patients often incur other expenditures, including payments for opportunistic infections (OIs) and non-ARV drugs as well as non-routine tests, medical consultations, transportation, food and hospital stays, that are seldom covered by any risk-pooling mechanism or government programme [5]. Non-ARV drugs include antibiotics, antivirals, blood tonics, and drugs for other OIs. Non-routine tests include x-rays, haemoglobin level and ultrasound.

Some studies have found that expenditures on treatment for those infected with HIV are potentially catastrophic, even within existing free ARV treatment programmes. These expenditures include substantial costs incurred for non-ARV drugs, non-routine tests, medical consultations and hospital stays, which match or sometimes exceed the costs of the ARV drugs alone [6–8]. There is evidence that expenditures incurred by households with people living with HIV/AIDS (PLHIV) on non-ARV-related costs, which are often borne through out-of-pocket payments, very often discourage people from using services or cause them to postpone health checks [9]. The high level of direct and indirect costs could lead to catastrophic health expenditures (CHEs) [10].

The evidence on the incidence of economic burden, especially occurrence of CHE on access to treatment for HIV/AIDS in the context of out-of-pocket spending, is still limited in many low- and middle-income countries, including Nigeria. Only a few studies have focused on the equity dimension of catastrophic health spending [11–15]. CHE occurs when out-of-pocket payments for healthcare exceed the estimated threshold share of household expenditure, over which a household is forced to sacrifice other basic needs, sell assets, incur debt or be impoverished [16,17]. The impact of CHE on households and individuals are well documented in literature [6,12,13,18–21]; apart from reducing utilization of health services, CHE can also lead to poverty or re-ranking of socio-economic status (SES) [22].

The existence of CHE signifies the failure of a health system to protect its citizens from financial consequences of healthcare [23]. Therefore, protection against CHE is considered a role that government should strive to perform [23,24]. Out-of-pocket payments are still the major payment mechanism for healthcare in Nigeria, as in many less developed and some developing countries. Evidence shows a positive correlation between the percentage of households caught in catastrophic payments and out-of-pocket-payments [25–29]. In addition, although CHE can occur for both the rich and poor, the consequences are often more disastrous for the poor, whose resources are limited [20].

As a means of characterizing the level of economic burden of diseases, several thresholds for measuring CHE have been proposed by different researchers in different settings [14], and the CHE thresholds have ranged from 5 to 40% of non-food expenditure [11]. However, it is important to use thresholds that are context-specific, since the levels of wealth across different geographic regions differ. Some authors used a threshold of 40% of *capacity to pay*, which was defined as “income after subsistence needs are met,” which in practice amounts to income minus food expenditure [11]. With more than two-thirds of Nigerians living below the US$1.25-a-day poverty head count ratio [30], any payment for health services in the country may be catastrophic and present a barrier to access [31].

This paper provides new information on the level of medical and non-medical expenditures incurred on outpatient visits (OPV) and inpatient admissions by patients in seeking treatment for HIV/AIDS. The paper also provides information and the resultant incidence of CHE from such expenditures to patients from different socio-economic groups and geographic locations. The information will increase the understanding of the level of expenditures borne by HIV/AIDS patients and will be important to guide policy and programmatic actions that will decrease the economic burden of HIV/AIDS. The findings of this study are expected to highlight potential areas for intervention that will ensure universal financial risk protection in access to holistic HIV/AIDS treatment services.

## Methods

### Study setting

The study was undertaken in three states selected from three geopolitical zones in Nigeria. The three states (Adamawa from the North-East zone, Akwa Ibom from the South-South zone and Anambra from the South-East zone) were chosen to obtain an approximately nationally representative view and to enable the estimation of the incidence of CHE in different geopolitical zones and geographic places of residence in Nigeria. In each state, an urban and rural local government area (LGA) were selected. In 2009 the prevalence of HIV was 3.8, 10.9 and 8.7% in Adamawa, Akwa Ibom and Anambra states, respectively. The 2006 census put the estimated population of the three states at 3,178,950; 3,902,051; and 4,177,828 for Adamawa, Akwa Ibom and Anambra states, respectively.

ARV therapy (ART) was fully subsidized in the three study states by the government and development partners. However, patients still paid for their laboratory investigations and any incident expenditures on co-morbidity. In Adamawa, some facilities also received fully subsidized treatment of OIs, whereas others charged a fee. In Akwa Ibom State, all patients were routinely charged a fee for treatment of OIs, whereas treatment for OIs was fully subsidized in Anambra State. Patients bore the costs of co-morbidities. Co-morbidities are incident illnesses that are not necessarily due to an individual being HIV positive. The common illnesses considered in this study were malaria and other febrile illnesses. OIs are those conditions that are likely due to diminished immunity of the individual suffering from HIV/AIDS.

### Study design

A descriptive cross-sectional household survey was undertaken from June to September 2013. A minimum sample size of 1200 was calculated using a power of 80 and 95% confidence interval, assuming a maximum catastrophic incidence level of 10%. However, in order to cover for refusals and incomplete data, the sample size was increased by 20% of the minimum calculated sample size. The target respondents were patients 18 years and above, living with HIV/AIDS. The patients were identified through support groups of the Association of PLHIV to avoid undue exposure of the HIV status of respondents, which could arise in a typical household survey. All eligible patients were included in the study, but the data on expenditures were from those patients who had been on ART and sought treatment within the three months preceding the survey. Informed consent was sought before determining patient's eligibility to participate in the survey.

### Data collection

Trained data collectors administered a pretested questionnaire to a sample of eligible respondents with HIV/AIDS. Patients were interviewed at home or at a location of their choice. Information was obtained on the most recent treatment sought by patients for HIV/AIDS within the three months preceding the survey. The questionnaire contained information on the demographic details of respondents, treatment seeking for HIV/AIDS (including outpatient and inpatient visits) and associated expenditures. The categories of expenditures included both medical expenditures and non-medical expenditures. The medical expenditures included expenses for laboratory tests, drugs, payment for hospital cards/registration and payments made before patient could be seen by a provider (consultation). The non-medical expenditures were expenditures such as transport, food and caregiver and accommodation expenses, where applicable. Information was also collected on weekly household food and non-food expenditures [5,14,32,33] and ownership of key household assets identical to those in the National Demographic and Health Survey conducted in Nigeria in 2008 [34] Annex 1.

### Data analysis

Data were analyzed from the patient's perspective for the whole sample. The main variables analyzed were patients’ demographic characteristics, medical and non-medical expenditures for outpatient and inpatient visits, and treatment-seeking behaviour. The components (sub-items) of medical expenditures were registration; consultation; and tests and drugs. Similarly, the components of non-medical expenditures were transport; food; accommodation; caregiver and others. The frequency distributions of categorical variables were calculated and the means calculated for non-categorical variables. Average costs were computed on the whole sample, since everybody incurred an expenditure in either one or all the expenditure components. The Kruskal–Wallis non-parametric test, which reports a chi-square statistic, was used to compare differences in means.

For estimating the SES, an SES index was developed using principal component analysis (PCA). The input into the PCA was information on households’ ownership of key assets, such as a car, electricity, radio, television, phone, fan, electric iron and so on, and *per capita* weekly household expenditures on food. The index was used to divide the individuals into five SES groups (quintiles), namely Q1 (poorest), Q2 (second), Q3 (third), Q4 (fourth) and Q5 (wealthiest). The chi-square for trend analysis was undertaken for all disaggregation of key dependent variables by SES quintiles. In addition, the equity ratio (Q1:Q5) was computed.

Data were disaggregated by SES and geographic location; mean and standard deviation were reported for the main outcome measures, which were the patient's medical and non-medical expenditures, and incidence of CHE were obtained and compared across urban-rural and SES groups. Significance was assessed at 5% (*p*-value < 0.05). The most recent outpatient or inpatient visit in the three months preceding the survey was used. Thus reported expenditures were for one inpatient or OPV, as the case may be. The three-month recall period was deemed appropriate to capture substantial inpatient events, given that they occur less frequently than OPVs.

The method proposed by Xu *et al*. [11] was used to estimate CHE, which by definition, refers to treatment-related expenditures exceeding 40% of a household's monthly non-food expenditure [11]. Other thresholds have been used in the literature [15]; however we explored two scenarios, which were monthly health expenditure as a share of monthly non-food expenditure greater than 40% and greater than 10% for inpatient and OPVs. These two thresholds were adopted in consideration of the high poverty levels in the context of the study, where more than 70% live on less than $1/day, and for the findings to be comparable to those in the literature. All expenditures are presented in US dollars using a 2014 exchange rate of US$1 to 160 Nigerian naira.

### Ethical considerations

All project staff completed the FHI 360 online ethics training before undertaking the surveys. Initial consent was obtained from the association of PLHIV in each location, and individual written consent was obtained before interviews were conducted, for those who volunteered to participate in the survey. Interviews were carried out discreetly to ensure minimal exposure of the respondents.

## Results

### Respondent characteristics

Data analysis was based on data from 1409 respondents with complete information. Table 1 shows that more than two-thirds (74%) of the respondents were females and 49.6% were monogamously married. The mean age of the respondents was 37. Overall, about 91% had some form of education. The average number of years spent in schooling was 10.6. The major source of income for a majority (45.2%) was petty trading. About half of the respondents (53%) were resident in rural areas. The average weekly expenditures and the *per capita* weekly expenditures on food were 6556.3 naira (US$41) and 1514.6 naira (US$9.5), respectively. The average monthly non-food expenditure was 10,926 naira (US$68.3).

**Table 1:** Demographic characteristics of respondents

Variable	*n* (%) *N* = 1409
Gender, *n* (%)	
Female	1048 (74.3)
Status in household, *n* (%)	
Male head	316 (22.4)
Female head	376 (26.7)
Son/daughter	228 (16.1)
Wife	489 (34.7)
Marital status, *n* (%)	
Married monogamous	699 (49.6)
Married polygamous	38 (2.7)
Single	262 (18.5)
Divorced	58 (4.1)
Separated	75 (5.3)
Widowed	275 (19.5)
Average number of all household residents, mean (SD)	5 (2.7)
Adults 18 +	3 (1.8)
13 to 17 years	1 (1.0)
12 and less	1.4 (1.4)
Age of respondents, mean (SD)	37 (9.9)
Attended school, *n* (%)	1279 (90.8)
Highest level of education, *n* (%)	
None	7 (0.5)
Primary education	400 (31.2)
JSS	149 (11.6)
SSCE	496 (38.8)
Tertiary	111 (8.7)
NCE	87 (6.8)
Other	30 (2.3)
Years spent schooling, mean (SD)	10.6 (3.8)
Major source of income, *n* (%)	
Unemployed	182 (12.9)
Farmer	128 (9.0)
Artisan/petty trader	638 (45.2)
Government worker	130 (9.3)
Self-employed	160 (11.3)
Employed in private sector	104 (7.3)
Other	66 (4.6)
Place of residence	
Urban	664 (47.1)
Rural	745 (52.9)
Weekly food expenditure, mean (SD)	6556.3 (3650.8)
*Per capita* weekly food expenditure, mean (SD)	1515 (992.0)
Average monthly non-food expenditure: mean (SD)	10,926 (10022.0)
SES distribution of respondents	
Quintile 1 (poorest)	282 (20.04)
Quintile 2 (second)	281 (19.97)
Quintile 3 (third)	282 (20.04)
Quintile 4 (fourth)	281 (19.97)
Quintile 5 (wealthiest)	281 (19.97)

SES, socio-economic status.

### Number of people that spent money on different items for OPV by SES and geographic place of residence

Analyses of expenditures were limited to 1392 respondents who had sought treatment for HIV/AIDS in the three months preceding the survey. It was found that 99% of respondents sought treatment in the three months preceding the survey. The most recent OPV was in the month directly preceding the survey for a majority of respondents across the three states (χ^2^ 119.6, *p*=0.00). More than two-thirds (73%) had been on ART for more than a year but frequency of check-up varied (χ^2^ 46.4, *p*=0.00). Overall, there were 35 admissions (less than 3% of respondents) within the three months preceding the survey, with an average of one admission in the period. Most admissions (51%) were in public facilities (χ^2^ 1.1, *p*=0.90), and more than two-thirds (77%) of admissions were for the treatment of OIs (χ^2^ 1.3, *p*=0.51) (not shown in table).

For the most recent OPV, the treatment received was mostly routine ARV drugs; for example over 90% of respondents in each state received routine treatment (χ^2^ 14.0, *p*=0.00). However, 79.4% of respondents in Anambra received treatment for other OIs, compared to 25 and 45% of respondents from Akwa Ibom and Adamawa states, respectively (χ^2^ 282.5, *p*=0.00).

Table 2 shows that majority of patients reported spending money on transport (97.8%) and food (72.8%). The proportions of respondents that spent money on other items are shown in Table 2.

**Table 2:** Proportion of people that spent money on different items for outpatient visit, by SES and geographic place of residence

	Outpatient visits									
	
Variables	Urban % *n* = 654	Rural % *n* = 738	Chi-square (*p*-value)	Q1 = poorest % *n* = 279	Q2 = second % *n* = 275	Q3 = third % *n*=277	Q4 = fourth % *n*=279	Q5 = wealthiest % *n*=280	Chi-square for trend (*p*-value)	Total *n* (%) *n* = 1392
Registration	20.8	42.9	76.8 (0.00)	36.9	28.8	30.9	32.4	33.0	0.34 (0.55)	452 (32.5)
Consultation	1.5	0.9	0.54 (0.45)	1.1	1.4	1.1	1.1	1.4	0.00 (0.93)	14 (1.0)
Tests	3.4	3.2	0.01 (0.90)	4.3	6.0	1.8	2.1	2.5	5.16 (0.02)	46 (3.3)
Drugs	13.3	6.7	16.59 (0.00)	9.1	7.1	8.2	13.5	11.0	2.89 (0.08)	136 (9.8)
Transport	97.1	97.4	3.39 (0.06)	96.8	95.0	95.7	97.2	98.2	0.52 (0.47)	1361 (96.6)
Food	71.8	70.9	0.26 (0.60)	69.5	75.1	69.9	73.3	69.0	0.34 (0.55)	1005 (71.3)
Accommodation	1.1	0	–	0.7	0.7	0.4	0	0.4	1.70 (0.19)	7 (.5)
Caregiver	1.2	2.3	1.72 (0.19)	4.3	1.1	1.4	1.8	0.4	8.61 (0.00)	25 (1.8)
Co-morbidities	6.6	11.1	7.74 (0.00)	8.1	9.6	9.6	10.3	6.8	0.35 (0.54)	124 (8.9)
Other expenditures	2.3	1.2	1.76 (0.18)	0.7	1.8	0.35	1.1	4.3	6.66 (0.00)	24 (1.7)

SES, socio-economic status.

### Average treatment expenditures for OPVs and inpatient stays

Table 3 shows that medical expenditures and non-medical expenditures contributed to 34.3 and 65.7%, respectively, of total expenditures for OPVs. Similarly, the table shows that medical expenditures and non-medical expenditures contributed to 38.7 and 61.3%, respectively, of total expenditures for inpatient stays (IPS). Table 3 also shows that for OPVs the main drivers for medical expenditures were the expenditures on drugs, at 271 naira (US$1.70), and tests, at 37 naira (US$0.22). Average non-medical expenditure was 647 naira (US$4.02). The most significant non-medical expenditure component was transport, with an average of 489 naira (US$3.05), followed by average expenditure on food, which was 144 naira (US$0.90). For IPS drug expenditure was the single most significant medical expenditure component, with an overall average of 4693 naira (US$29.30).

**Table 3:** Average treatment expenditures for outpatient visits and inpatient stays

Variable	Outpatient visits Mean total in naira (US$)	Inpatient stays Mean total in naira (US$)	Percentage of expenditure components of outpatient visits in total expenditure	Percentage of expenditure components of in-stays in total expenditure
Medical expenditures	338 ($2.11)	5712 ($35.70)	34.3	38.7
Registration	28 ($0.17)	181 ($1.13)	2.7	1.2
Consultation	3 ($0.02)	183 ($1.14)	0.3	1.2
Tests	37 ($0.22)	656 ($4.10)	3.8	4.4
Drugs	271 ($1.70)	4693 ($29.30)	27.5	31.8
Non-medical expenditures	647 ($4.02)	9055.4 ($56.59)	65.7	61.3
Transport	489 ($3.05)	833 ($5.20)	49.6	5.6
Food	144 ($0.90)	2291 ($14.32)	14.9	15.5
Accommodation	2 ($0.01)	2183 ($13.64)	0.2	14.8
Caregiver	4 ($0.02)	806 ($5.03)	0.4	5.5
Other expenditures	5 ($0.03)	2943 ($18.39)	0.5	19.9
Average total expenditure	985 ($6.10)	14,767.0 ($92.30)	100	100

*Note:* US$1 = 160 Nigerian naira.

### Differences in average outpatient expenditures by SES and geographic location

The result in Table 4 is based on OPVs, since there were few people that incurred inpatient expenditures. There were significant urban-rural differences in average medical and non-medical expenditures per visit for HIV/AIDS treatment (*p*<0.05), and across SES and urban-rural residence, non-medical expenditures (US$4.00) were about twice the medical expenditures (US$2.10). For medical expenditures the urban dwellers paid more (US$2.20 compared to US$2.00), whereas the reverse was true for non-medical expenditures. The expenditures on treatment of co-morbidities was more than 50% of total medical expenditures with significant urban-rural differences (*p* = 0.00). The expenditures on food (US$0.90) and transport (US$3.00) were much higher than the other categories of non-medical expenditures, and they contributed to 15 and 50% of total outpatient expenditures on HIV/AIDS treatment, respectively. For transport, the rural dwellers paid more (US$3.40, *p*=0.00).

**Table 4:** Differences in average outpatient expenditures by SES and geographic location

Variables	Total *N=*1392 Mean (SD)	Rural Mean (SD) N=654	Urban Mean (SD) *N=*738	Chi-square (*p*-value)	Q1=poorest *N=*279	Q2=second *N*=275	Q3=third *N*=277	Q4=fourth *N*=279	Q5=wealthiest *N*=280	Chi-square (*p*-value)	Q1:Q5 ratio (equity ratio)
Medical expenditures	2.1 (6.6)	2.0 (6.2)	2.2 (6.9)	5.9 (0.01)	1.7 (7.2)	2.5 (7.2)	2.2 (7.9)	1.8 (4.5)	2.4 (7.7)	1.98 (0.73)	0.7
Registration	0.2 (0.4)	0.2 (0.3)	0.0 (0.4)	41.2 (0.00)	0.1 (0.3)	0.2 (0.4)	0.1 (0.3)	0.2 (0.4)	0.2 (0.4)	1.61 (0.81)	0.5
Consultation	0.02 (0.3)	0.0 (0.0)	0.0 (0.4)	0.0 (0.84)	0.0 (0.0)	0.0 (0.3)	0.0 (0.2)	0.0 (0.2)	0.0 (0.4)	0.01 (1.00)	0
Tests	0.2 (1.7)	0.2 (1.8)	0.2 (1.5)	0.0 (0.93)	0.2 (1.3)	0.4 (1.7)	0.1 (1.1)	0.1 (1.5)	0.3 (2.4)	1.12 (0.89)	0.6
ARV drugs	0.6 (2.6)	0.4 (2.4)	0.9 (2.8)	4.6 (0.00)	0.5 (2.3)	0.5 (2.3)	0.5 (2.4)	0.7 (2.6)	0.9 (3.4)	1.96 (0.74)	0.6
Co-morbidities	1.1 (5.4)	1.1 (5.1)	1.0 (5.7)	7.9 (0.00)	0.8 (3.9)	1.4 (6.2)	1.4 (7.2)	0.7 (2.5)	1.0 (6.0)	0.66 (0.95)	0.8
Non-medical expenditures	4.0 (4.1)	4.2 (4.3)	3.8 (3.9)	9.0 (0.00)	3.9 (3.7)	3.8 (3.0)	4.1 (4.6)	3.6 (3.1)	4.6 (5.7)	5.39 (0.24)	0.8
Transport	3.0 (3.8)	3.4 (4.1)	2.7 (3.3)	28.8 (0.00)	3.1 (3.4)	2.8 (2.7)	3.3 (4.4)	2.6 (2.7)	3.5 (4.9)	5.72 (0.22)	0.9
Food	0.9 (1.0)	0.8 (0.8)	0.9 (1.2)	2.2 (0.14)	0.7 (1.0)	0.9 (1.0)	0.8 (0.7)	1.0 (1.0)	1.0 (1.2)	11.99 (0.01)	0.7
Accommodation	0.0 (0.2)	0	0.0 (0.3)	0.1 (0.73)	0.0 (0.1)	0.0 (0.1)	0.0 (0.0)	0 (0)	0.0 (0.4)	0.03 (0.99)	0
Caregiver	0.02 (0.2)	0.03 (0.3)	0.0 (0.3)	0.1 (0.73)	0.1 (0.5)	0.0 (0.1)	0.0 (0.2)	0.0 (0.2)	0.0 (0.1)	0.75 (0.94)	0.1
Other expenditures	0.03 (0.5)	0.0 (0.3)	0.0 (0.6)	0.1 (0.73)	0.0 (0.1)	0.0 (0.1)	0.0 (0.0)	0.0 (0.1)	0.1 (1.0)	0.83 (0.93)	0
Total OPV expenditures	6.1 (8.0)	6.3 (8.1)	5.9 (7.9)	5.9 (0.01)	5.6 (6.6)	6.3 (8.0)	0.6 (9.6)	5.3 (5.5)	7.1 (7.1)	1.98 (0.73)	0.7

Note: The median for total expenditures on outpatient visit was $3.90 and the range was $0 to $100.10.

SES, socio-economic status; OPV, outpatient visit.

Across SES, there were no significant differences in expenditure categories except for those on food (*p*=0.01), where those in the higher SES spent more. The rural-urban equity ratios show that medical expenditures were pro-poor (0.91), whereas for non-medical costs the rural dwellers paid more (1.11). The ratios for all expenditure categories across SES suggest that the poor pay less compared to the rich except for caregiver expenditures. Few respondents paid for consultation and the result is not significantly different across SES and the rural-urban divide (Table 4). The median for total expenditures on OPVs was US$3.90 and the range was $0 to $100.10.

### SES and geographic differences in level of CHEs

Table 5 shows that overall, at a threshold of 40%, about 8 and 94% of patients incurred catastrophic expenditures on outpatient and inpatient visits, respectively. At a threshold of 10%, the corresponding numbers were 40 and 100%, respectively. At the 40% threshold, rural dwellers (*p*=0.00) and the poorest quintile (*p*=0.00) were more likely to incur catastrophic expenditures for OPVs; there were no significant differences for inpatient visits. At the 10% threshold for OPVs, again rural dwellers (*p*=0.00) and the poorest (*p*=0.00) were significantly more likely to incur catastrophic costs. For inpatient visits at the 10% threshold, everyone incurred catastrophic expenditure. There were no significant differences in catastrophic expenditures by state (Table 5).

**Table 5:** Differences in the level of catastrophic health expenditures in different population groups

	Total outpatient expenditure >40% of non-food expenditure, *n* (%)	Total inpatient expenditure >40% of non-food expenditure, *n* (%)	Total outpatient expenditure >10% of non-food expenditure, *n* (%)	Total inpatient expenditure >10% of non-food expenditure, *n* (%)
Combined	107 (7.7)	33 (94.3)	561 (40.3)	35 (100)
Urban-rural differences				
Urban	37 (5.7)	10 (90.9)	188 (28.7)	11 (100.0)
Rural	70 (9.5)	23 (95.8)	373 (50.5)	24 (100.0)
Chi^2^ (*p*-value)	7.20 (0.00)	0.33 (0.53)	68.8 (0.00)	
Socio-economic status differences				
Poorest	42 (15.0)	10 (100.0)	161 (57.7)	10 (100.0)
Second	20 (7.3)	4 (80)	126 (45.8)	5 (100)
Third	22 (7.9)	6 (100)	115 (41.5)	6 (100)
Fourth	11 (3.9)	6 (100)	87 (31.1)	6 (100)
Wealthiest	12 (4.3)	7 (80)	72 (25.7)	8 (100)
Chi^2^ (*p*-value)	31.5 (0.00)	3.91 (0.34)	73.4 (0.00)	
Differences by state				
Adamawa	396 (92.1)	17 (80.9)	175 (40.7)	20 (95.2)
Akwa Ibom	426 (93.6)	4 (100.0)	173 (38.0)	4 (100.0)
Anambra	463 (91.3)	10 (100.0)	213 (42.0)	10 (100.0)
Chi^2^ (*p*-value)	1.84 (0.39)	1.63 (0.44)	1.17 (0.55)	0.68 (0.71)

## Discussion

The findings show that the public provision of free drugs is not enough to eliminate the high and sometimes inequitable economic burden of HIV/AIDS on households. Many adjunct treatments and expenditures on diagnostics that are not covered by free ART programmes still predispose patients to incurring CHE. In addition, some non-medical expenditures that are incurred by patients, such as transport and feeding during treatment visits, are also substantial contributors to the high level of economic burden of HIV/AIDS to households. Inpatient visits particularly led to a high level of CHE.

Irrespective of the free provision of ARV drugs at several facilities across Nigeria, patients still need to pay out-of-pocket for other medical expenditures, such as OIs and co-morbidities, as well as non-medical expenditures. Other studies have similarly reported that treatment seeking could still remain unaffordable despite the availability of free ARVs due to other care components associated with HIV/AIDS [6,7]. In Ghana, total outpatient expenditure on ART was found to be up to US$55 depending on how far the patients had to travel to get to the nearest ART centre and how long they had to wait at the ART facility [7]. Similar findings were reported by other studies elsewhere [8,35,36]. Rosen *et al*. found that 91% of patients paid for transport to attend ART clinics and 60% of patients purchased non-prescription medicines or special food at considerable cost [35]. It was also found in Kenya that patients made an average payment of US$7 for ART [36]. These findings were similar to the high incidence and levels of expenditures on transport that was found in our study.

It was revealing to find that non-medical expenditures were much higher than medical expenditures and that the two most significant expenditure components were food and transport to treatment facilities. Hence, it does appear that policy interventions such as decentralizing treatment centres by bringing them nearer to people that can significantly lower these expenditures to PLHIV and their households. Such measures will subsequently improve health seeking for PLHIV and adherence to ART [37]. In the long term, they are expected to significantly improve the welfare of the patients and households. Moon *et al*. show that expenditures for transport may pose significant barriers of access to ARVs even where they are free [6].

The findings show that rural dwellers, who are usually poorer than the urbanites as reflected by their lower SES, suffered greater economic burden in accessing ART services and treatment for other HIV/AIDS-related conditions. Overall, there were geographic differences in medical and non-medical expenditures. The average treatment expenditure for an OPV was higher for the rural dwellers, but it was not clear why there was no difference in some of the expenditures across the three states. It is possible that the states are not programmatically different from each other. However, this line of inquiry could be a subject for future studies.

It was found that the households’ expenditures on HIV/AIDS were to a large extent catastrophic and that the magnitude was significantly higher for the rural dwellers and those from lower SES. The magnitude of CHE in the study was less than reported in a previous study, which found quite high levels of CHE for patients regardless of geographic locations, sex or SES [5], possibly due to increases in the number of ART facilities in the region. However, it should be noted that more than 70% of Nigerians live below the poverty line and they usually spend all their money on food; any other expenses are potentially catastrophic. Hence, the use of 40% or even 10% non-food expenditure thresholds may be misleading in the context of the study. Non-availability of money when a person needs healthcare is a major barrier to accessing healthcare services in Nigeria [4].

The greater differences in expenditures and incidence of CHE by geographic location of the households rather than by SES implies that rural-urban inequity is a more significant problem than SES differences in Nigeria within the context of treatment for HIV/AIDS. This inequity is addressable, but the intervention may have to adopt a multisectoral approach to address the multifaceted problems impoverishing PLHIV and their households. Such an approach may involve the development and implementation of some income-generating interventions. Policy options could be explored to support the provision of a full subsidy for payment of OIs. It is not easy to understand the non-difference in CHE between the states. However, it is possible that since all the states are guided by the national guidelines of ART provision, they are not expected to be programmatically different at the macro level.

Our findings confirm that hospitalization greatly increases the possibility of incurring catastrophic medical expenditures among households. Although the proportion of people having inpatient visits was low, almost all expenditures were catastrophic regardless of SES group and place of residence when the threshold was lowered to 10% and more than half at 40%. The finding that OIs account for most hospitalizations is in line with studies elsewhere that have demonstrated the role of OIs in HIV/AIDS morbidity and mortality [38]. In less-developed countries where there are limited risk-pooling mechanisms to cover individuals from medical expenditures, hospitalizations are associated with high levels of expenditures [39], especially when they occur at private facilities where patients receive few or no subsidies for treatment.

A limitation of the study was the restriction of survey respondents to those who belonged to a support group for PLHIV. This implies that the sample eliminated people that did not belong to the groups and was potentially self-selected. However, recruiting respondents from only support groups was done to avoid exposing the HIV status of respondents if they were selected from a random household survey. Another limitation was that data on frequency of visits were not collected, since some patients visit clinics often within one month, and such multiple visits may lead to underestimation of CHE if not captured. Moreover, there was no qualitative component that could have been used for deeper exploration of some of the issues and no external validation of expenditure data from other sources. These could be researched in future studies. Furthermore, future studies could explore the impact of different funding arrangements on catastrophic expenditures. Finally, relying on patient's recall of expenditures on services may have affected the accuracy of information and therefore represents a potential source of bias in this study [40]. An alternative approach could be to explore the differences in cost of treatment for patients on ART versus those not on ART.

## Conclusions

All in all, households’ expenditures for their members that are living with HIV/AIDS (PLHIV) to receive treatment for the disease was quite high, inequitable and catastrophic in some instances, hence further jeopardizing the welfare of the household as a whole, as well as the PLHIV. The fact that a greater share of treatment expenditures were from the transport and food expenditure categories suggests that locating treatment centres closer to PLHIV and deploying more health personnel to the treatment centres will reduce travel expenditures, improve adherence to treatment and lessen the need to spend a lot of time during care visits, which necessitates expenditures on feeding at the ART facilities. In addition, financial risk-protection mechanisms should be implemented that will significantly eliminate the expenditures borne by PLHIV and their households in order to receive ART services. In particular, subsidies of expenditures on transport in the form of vouchers or reimbursement systems are good financial protection mechanisms. Enhancing the income of PLHIV and their households can reduce the incidence of CHE, since CHE increased as SES decreased. Finally, universal financial risk protection within the sphere of universal health coverage should be the ultimate goal of HIV/AIDS treatment services, so as to protect all households against CHEs.

## Figures and Tables

**Annex 1:**
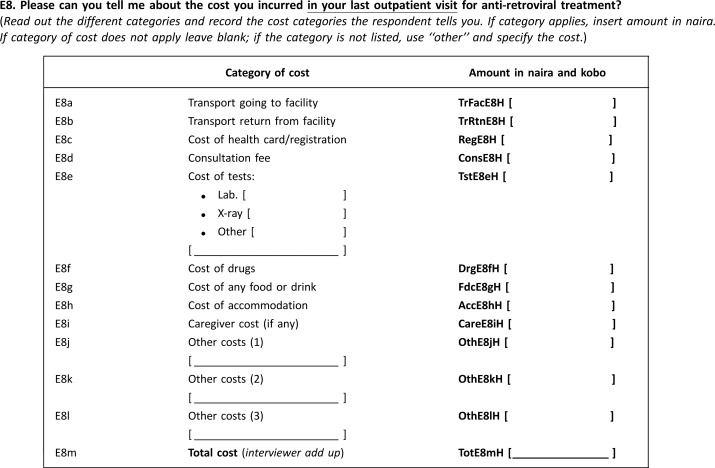
Sample questions used to collect expenditure data
